# Case Report: Aumolertinib as Neoadjuvant Therapy for Patients With Unresectable Stage III Non-Small Cell Lung Cancer With Activated EGFR Mutation: Case Series

**DOI:** 10.3389/fonc.2022.872225

**Published:** 2022-03-29

**Authors:** Shao Feng, Zhang Qiang, Cheng Wanwan, Zeng Zhaozhun, Xie Yuewu, Fang Shencun

**Affiliations:** ^1^ Department of Thoracic Surgery, Nanjing Chest Hospital, The Affiliated Brain Hospital of Nanjing Medical University, Nanjing, China; ^2^ Department of Respiratory Medicine, Nanjing Chest Hospital, The Affiliated Brain Hospital of Nanjing Medical University, Nanjing, China; ^3^ Medical Oncology Scientific Group of the Central Medical Department, Jiangsu Hansoh Pharmaceutical Group Co., Ltd., Shanghai, China

**Keywords:** aumolertinib, NSCLC, neoadjuvant, EGFR, adjuvant

## Abstract

**Background:**

There is no standard treatment for stage III lung cancer due to its low surgical resection rate, and improving PFS and survival of patients with III NSCLC has become an urgent challenge in clinical treatment. For EGFR mutation-positive patients, targeted therapy has the remarkable feature of high efficiency and low toxicity compared with first-line standard chemotherapy, and targeted neoadjuvant therapy needs to be further explored.

**Method:**

We report 3 diagnosed cases of locally advanced unresectable NSCLC with EGFR-sensitive mutations who first received 1–2 cycles of preoperative chemotherapy neoadjuvant therapy and were treated with 110 mg daily of 3rd-generation EGFR-TKI aumolertinib instead because of poor efficacy or safety intolerance.

**Result:**

After 2 cycles of aumolertinib treatment, all 3 patients achieved symptomatic remission and significant tumor size reduction and achieved downstaging to allow surgical treatment. No additional operative difficulties were added during the surgery. They continued to receive adjuvant therapy with the original dose of aumolertinib after surgical treatment, and no evidence of tumor recurrence was found until the most recent imaging examination. In addition, the course of neoadjuvant and adjuvant therapy was free of serious adverse effects.

**Conclusion:**

Perioperative treatment of these three cases of locally advanced unresectable NSCLC with EGFR-sensitive mutations with the third-generation EGFR-TKI aumolertinib showed significant efficacy and excellent safety and may be a new option for targeted therapy in the perioperative period.

## Introduction

Patients with stage III non-small cell lung cancer (NSCLC) have considerable disease heterogeneity (1, 2). Treatment options include neoadjuvant therapy followed by surgical resection, adjuvant chemotherapy after surgery, or radical radiotherapy. A large meta-analysis confirmed the survival benefit of preoperative chemotherapy (3). However, the lung damage caused by preoperative chemotherapy may also make subsequent surgical resection more difficult (4, 5). No consensus exists with regard to optimal treatment approaches, particularly for unresectable advanced NSCLC characterized by single-site or multisite lymph node metastases identified by preoperative staging; the role of surgical resection after preoperative therapy remains controversial (6–8).

Epidermal growth factor receptor (EGFR) tyrosine kinase inhibitors (TKI) significantly prolong progression-free survival (PFS) in patients with advanced EGFR mutation-positive (EGFRm) NSCLC compared to chemotherapy as first-line therapy (9–11). Aumolertinib (HS-10296), a third-generation EGFR-TKI, has attracted much attention due to its reliable antitumor activity and favorable safety profile (12–14).

Although EGFR-TKI was established as an effective first-line therapy for advanced EGFRm NSCLC (9–11), the efficacy and tolerability of EGFR-TKI preoperative induction therapy remain uncertain. We report three cases of stage III NSCLC with confirmed EGFR mutations, who reached a descending stage after preoperative neoadjuvant treatment with aumolertinib and had successful surgical resection of the malignant tumor.

## Presentation

### Case 1

**Figure 1:**
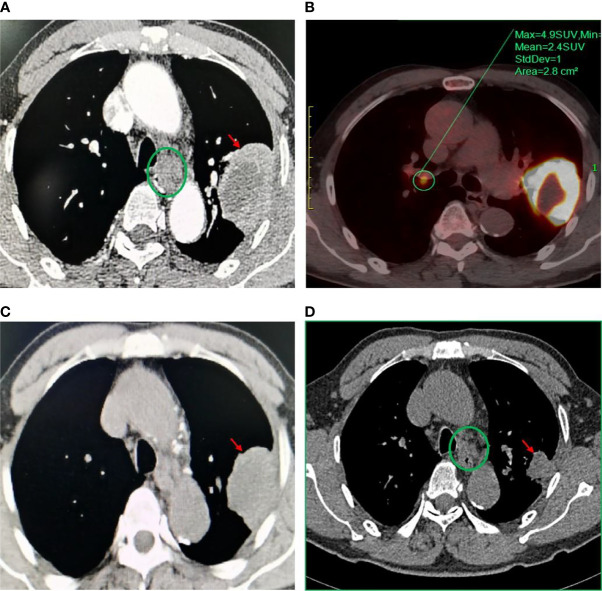
Disease status before and after neoadjuvant chemotherapy and aumolertinib treatment. Computed tomography chest (CT) scan showed **(A)** pulmonary nodule in the left upper lobe of the lung, size 7.8 × 4.9 cm before chemotherapy. PET/CT showed **(B)** lymph node enlargement in the right hilar and pulmonary nodule in the left upper lobe before chemotherapy. **(C)** Stable disease (SD) based on imaging findings after 2 cycles of chemotherapy. **(D)** Pulmonary nodule in the left upper lobe of the lung, size 2.2 × 2.0× 1.8 cm after almost 2 months of aumolertinib treatment, with tumor remission reaching partial remission (PR).

A 64-year-old Chinese male, a 40-year smoker and hypertension, presented to a local hospital in January 2021 with an irritating cough and sputum with blood in the sputum, and a computed tomography chest (CT) scan showed a 7.8 × 4.9-cm left upper lung space. Lung puncture biopsy showed squamous carcinoma, and immunohistochemistry showed high PD-L1 expression, TPS ≥ 90%, EGFR mutation (exon 19 deletion) by NGS, and clinical stage IIIC (T3N3M0). PET/CT showed left lung cancer with obstructive pneumonia, enlarged lymph nodes in the left clavicular region, posterior to the anterior vena cava, aortic window, left paratracheal area, and both hila, and increased FDG metabolism. Magnetic resonance imaging (MRI) of the brain showed no evidence of central nervous system (CNS) involvement, which made her malignancy compatible. **Figure 1** shows the imaging findings of case 1.

Systemic chemotherapy was started in January 2021 with an initial dose of albumin-bound paclitaxel 400 mg and carboplatin 0.4 mg. Rash and pruritus developed during the second chemotherapy infusion of carboplatin, so treatment with carboplatin was discontinued. After 2 cycles of chemotherapy, objective tumor remission was evaluated as disease stabilization (SD) by RECIST 1.1 (efficacy evaluation criteria for solid tumors). After nearly 8 weeks of oral administration of the third-generation EGFR-TKI aumolertinib (110 mg/day) started in February 2021, imaging showed significant tumor regression, lesion volume reduction to 2.2 × 2.0 × 1.8 cm, partial remission (PR), and clinical stage reduction to stage IB (T2N0M0). After evaluation by the surgeon, the patient was eligible for surgery and underwent VATS left upper lung lobectomy + lymph node dissection with the patient’s consent in April 2021. Postoperative pathology showed interstitial fibrous lung tissue with numerous lymphocytic infiltrates, with large necrotic tissue and <10% residual tumor cells (major pathological response, MPR), which was considered as posttreatment changes of squamous carcinoma. There was no metastasis of cancer tissue in any of the lymph nodes examined. After discussion with the patient, he was willing to receive adjuvant therapy with aumolertinib, and the disease was judged to be stable by imaging results in February 2022.

### Case 2

**Figure 2:**
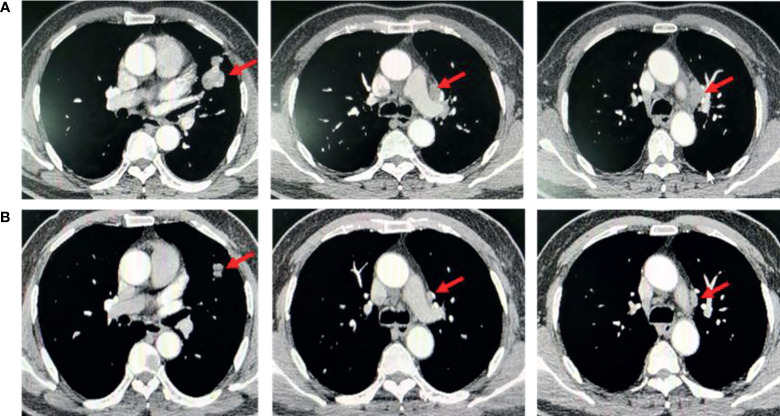
Disease status before and after neoadjuvant almonertinib therapy. Computed tomography chest (CT) scan showed **(A)** left lingual lobe occupancy with a size of 4.0 × 3.8 × 2.3 cm, and subaortic lymph nodes before aumolertinib treatment. **(B)** Left lingual lobe occupancy with a size of 1.2 × 1 × 1.5 cm, and subaortic lymph nodes after aumolertinib treatment.

A 68-year-old Chinese male with grade 3 hypertension was admitted to the hospital in December 2020 with a 1-week history of pulmonary occupancy found on physical examination. Chest CT revealed a left lingual lobe occupancy with obstructive pneumonia and enlarged mediastinal and left axillary lymph nodes; the size of the tumor was 4.0 × 3.8 × 2.3 cm, and cranial MRI, abdominal ultrasound, and bone scan was unremarkable. Lung puncture biopsy was diagnosed as adenocarcinoma, and immunohistochemistry showed a negative PD-L1 expression with TMB: 6.38 mut/bp; NGS revealed that the patient had EGFR exon 21 L858R point mutation, combined with HEBB2 amplification, EGFR amplification, and TP53 mutation; and the clinical stage was IIIA (T2N2M0). The patient’s enlarged lymph node in the left hilum was completely fused with the left pulmonary artery trunk and could not be completely removed surgically, which was an unresectable stage III lung cancer. After 2 cycles of pemetrexed combined with carboplatin chemotherapy in December 2020, the tumor did not shrink significantly according to chest CT. Considering that the patient had EGFR 21 exon L858R mutation, she was treated with oral aumolertinib 110 mg/day in January 2021, and after nearly 2 months of treatment, chest CT indicated significant regression of the left hilar lymph nodes, and the mass volume was reduced to 1.2 × 1.0 × 1.5 cm, achieving PR; clinical stage was reduced to stage IB (T2N0M0); and the tumor was clearly demarcated from the left pulmonary artery trunk, which could be considered for surgical radical treatment. **Figure 2** shows the imaging findings of case 2.

Therefore, VATS left upper lung lobectomy and lymphatic dissection were performed on February 22, 2021. Postoperative pathology showed moderately differentiated adenocarcinoma with elastic plaque formation, tumor involvement in 0/10 lymph nodes, and residual tumor cells <10% (MPR). The postoperative pathological stage was stage IA (T1N0M0). After surgery, the patient had been receiving adjuvant therapy with aumolertinib for 11 months, and no evidence of malignancy recurrence was seen on spiral CT of the chest.

### Case 3

**Figure 3:**
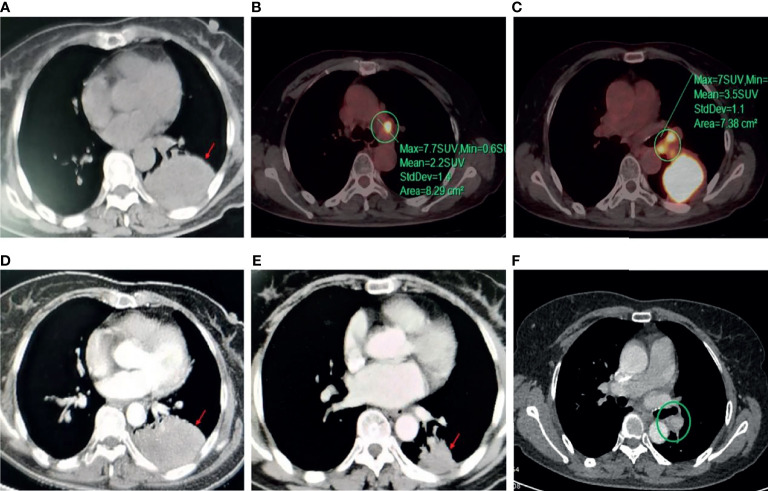
Disease status before and after neoadjuvant chemotherapy and aumolertinib treatment. Computed tomography chest (CT) scan showed that **(A)** before chemotherapy, the size of the left lower lung soft tissue mass was 10.9 × 8.9 × 6.5 cm. PET/CT showed **(B)** mediastinal lymph node enlargement before chemotherapy. **(C)** Multiple lymph node enlargement in the left hilar before chemotherapy. CT scan showed that **(D)** after 2 cycles of chemotherapy, the disease was judged to be stable based on imaging findings. **(E)** After 2 cycles of aumolertinib treatment, the size of the left lower lung soft tissue mass was 3.0 × 4.0 × 4.0 cm, and the tumor achieved partial remission. **(F)** After 2 cycles of aumolertinib treatment, multiple lymph nodes disappeared in the left hilar.

A 60-year-old Chinese female with grade 2 hypertension was admitted with cough for 1 month. Chest CT showed a left lower lung occupancy with pulmonary atelectasis; PET/CT showed a left lower lung soft tissue mass (10.9 × 8.9 × 6.5 cm), left lower lobe bronchial truncation, aortic window, and multiple lymph node enlargement in the left hilar, and left lung cancer with obstructive pneumonia was considered. Lung puncture biopsy and immunohistochemistry diagnosed squamous carcinoma; immunohistochemistry results showed negative PD-L1 expression, and NGS test results were EGFR L858R mutation combined with TP53 mutation, clinical stage IIIB (T4N2M0). Thoracic surgery specialists consulted and judged that there was no indication for surgery for the time being and recommended medical treatment. **Figure 3** shows the imaging findings of case 3.

The patient was treated with a chemotherapy regimen of albumin paclitaxel combined with carboplatin for two cycles in November 2020, and a chest CT review showed that the lesion was still stable, but the tumor did not shrink significantly. Therefore, the patient received oral aumolertinib (110 mg/day) for 2 cycles from January 2021, and the tumor shrank significantly from before to 3.0 × 4.0 × 4.0 cm, reaching PR and clinical stage down to stage IB (T2N0M0), which was eligible for surgery. Therefore, the patient requested surgical treatment and underwent VTAS left lower lung lobectomy, lymph node dissection, and pleural adhesion release in March 2021. Postoperative pathology showed focal fibrous tissue hyperplasia with inflammatory cell infiltration in lung tissue, focal bronchial epithelial adenoid formation, and mild alveolar epithelial cell hyperplasia; some multinucleated giant cell reaction was seen; posttreatment changes were considered; no tumor residual was seen; and the status of pathological complete response (pCR) was achieved. 0/10 lymph nodes had tumor involvement, and the postoperative pathological stage was stage 0 (T0N0M0). After surgery, the patient was treated with aumolertinib for adjuvant therapy, and the disease was judged to be stable on the chest CT findings in January 2022.

## Discussion

Three patients with unresectable stage III non-small-cell lung cancer were first treated with chemotherapy and were switched to aumolertinib for 2 cycles due to poor efficacy, and all achieved downstaging and met resectability criteria after neoadjuvant aumolertinib treatment. Postoperative pathology showed MPR in the first two cases and pCR in the last case. In addition, the safety profile of aumolertinib in neoadjuvant attempts was satisfactory, with no events that delayed surgery or increased surgical complications. Patient characteristics and treatment response before and after surgery are shown in **Table 1**.

**Table 1:** Patient characteristics and response to treatment before and after surgery (N = 3).

Patient	Gender/age	Histological types	EGFR mutation subtypes	PD-LI expression status	First-line treatment		Second-line treatment		Pathologicalassessment
					Treatment	Response	Treatment	Response
1	M/64	Squamous carcinoma	exon19 deletion	Positive	Chemotherapy	SD	Aumolertinib	PR	MPR
2	M/68	Adenocarcinoma	L858R mutation	Negative	Chemotherapy	SD	Aumolertinib	PR	MPR
3	F/60	Squamous carcinoma	L858R mutation	Negative	Chemotherapy	SD	Aumolertinib	PR	pCR

SD, stable disease; PR, partial response; MPR, major pathological response; pCR, pathological complete response.

In this case series report, the first patient who received chemotherapy had a more severe rash and pruritus, which the physician judged to be a hypersensitivity reaction caused by carboplatin, which has been shown to predispose patients treated with carboplatin in combination with paclitaxel (15). Symptomatic treatment was replaced with oral aumolertinib and continued for 8 weeks, with imaging showing partial remission and no serious adverse events.

The second and third cases also received 2 cycles of preoperative chemotherapy without significant tumor remission. After chemotherapy and switching to oral aumolertinib treatment for 4–8 weeks with the patient’s consent, imaging showed that both patients responded well to treatment, with significant tumor volume reduction to a descending stage that allowed them to undergo surgery.

This may indicate an efficacy advantage of aumolertinib in the neoadjuvant phase of therapy. Similarly, results from the EMERGING-CTONG 1103 study showed a trend toward improved ORR, lymph node step-down, MPR, and R0 resection rates with neoadjuvant erlotinib compared to neoadjuvant chemotherapy for patients with EGFRm NSCLC, and significantly prolonged PFS (16). Evidence from case reports and small non-randomized clinical trials reported suggests that neoadjuvant EGFR-TKI therapy is potentially efficacious in patients with resectable NSCLC (17–21). This suggests that neoadjuvant-targeted therapy modalities are potentially clinically applicable and may be a viable option for patients who are not optimal chemotherapy candidates due to medical comorbidities or who refuse chemotherapy.

Finally, overall oncologic outcomes may be influenced by the timing of surgical intervention after neoadjuvant therapy. A large retrospective study found that in patients with stage IIIa NSCLC, 1- and 3-year survival rates were significantly lower in the short-delayed group after preoperative neoadjuvant therapy compared with the long-delayed group (22). Due to the significant safety advantage of targeted agents over chemotherapy, preoperative EGFR-TKI neoadjuvant therapy may reduce lung injury and make surgery less difficult. Therefore, it may shorten the time between the end of neoadjuvant therapy and surgery, thus affecting prognostic survival. Currently, the optimal duration of preoperative induction-targeted therapy is unclear, and whether 6 weeks of EGFR-TKI induction therapy is sufficient has yet to be verified, so there may be a problem of insufficient induction-targeted therapy.

Aumolertinib has demonstrated excellent tumor remission and a favorable safety profile in the neoadjuvant and adjuvant phases of these three phase III NSCLC cases, which has implications in guiding targeted therapy in the perioperative setting of NSCLC. However, its ability to be a firm choice for neoadjuvant and adjuvant treatment of NSCLC still needs to be validated by large randomized controlled studies.

## Data Availability Statement

The original contributions presented in the study are included in the article/supplementary material. Further inquiries can be directed to the corresponding author.

## Ethics Statement

The studies involving human participants were reviewed and approved by the research ethics committee of the Affiliated Brain Hospital of Nanjing Medical University. The patients/participants provided their written informed consent to participate in this study.

## Author Contributions

All authors contributed toward data analysis, drafting, and critically revising of the paper, gave final approval of the version to be published, and agree to be accountable for all aspects of the work.

## Conflict of Interest

Authors ZZ and XY are employed by Jiangsu Hansoh Pharmaceutical Group Co., Ltd.

The remaining authors declare that the research was conducted in the absence of any commercial or financial relationships that could be construed as a potential conflict of interest.

## Publisher’s Note

All claims expressed in this article are solely those of the authors and do not necessarily represent those of their affiliated organizations, or those of the publisher, the editors and the reviewers. Any product that may be evaluated in this article, or claim that may be made by its manufacturer, is not guaranteed or endorsed by the publisher.

## References

[1] van Meerbeeck JP Surmont VF. Stage IIIA-N2 NSCLC: A Review of its Treatment Approaches and Future Developments. Lung Cancer (2009) 65:257–67. doi: 10.1016/j.lungcan.2009.02.007 19285751

[2] Pang Z Yang Y Ding N Huang C Zhang T Ni Y. Optimal Managements of Stage IIIA (N2) Non-Small Cell Lung Cancer Patients: A Population-Based Survival Analysis. J Thorac Dis (2017) 9(10):4046–56. doi: 10.21037/jtd.2017.10.47 PMC5723778 29268415

[3] NSCLC Meta-analysis Collaborative Group. Preoperative Chemotherapy for Non-Small-Cell Lung Cancer: A Systematic Review and Meta-Analysis of Individual Participant Data. Lancet (2014) 383(9928):1561–71. doi: 10.1016/S0140-6736(13)62159-5 PMC4022989 24576776

[4] Pisters KMW Vallières E Crowley JJ Franklin WA Bunn PA Jr Ginsberg RJ. Surgery With or Without Preoperative Paclitaxel and Carboplatin in Early-Stage Non-Small-Cell Lung Cancer: Southwest Oncology Group Trial S9900, an Intergroup, Randomized, Phase III Trial. J Clin Oncol (2010) 28(11):1843–9. doi: 10.1200/JCO.2009.26.1685 PMC2860367 20231678

[5] Depierre A Westeel V. Preoperative Chemotherapy in Non-Small Cell Lung Cancer: Advantages, Disadvantages, Level of Evidence. Rev Mal Respir (2007) 24:6S59–63. doi: 10.1019/200720130 18235395

[6] Rusch VW Giroux DJ Kraut MJ Crowley J Hazuka M Winton T. Induction Chemoradiation and Surgical Resection for Superior Sulcus Non-Small-Cell Lung Carcinomas: Long-Term Results of Southwest Oncology Group Trial 9416 (Intergroup Trial 0160). J Clin Oncol (2007) 25:313–8. doi: 10.1200/JCO.2006.08.2826 17235046

[7] Darling GE Li F Patsios D Massey C Wallis AG Coate L. Neoadjuvant Chemoradiation and Surgery Improves Survival Outcomes Compared With Definitive Chemoradiation in the Treatment of Stage IIIA N2 Non-Small-Cell Lung Cancer. Eur J Cardiothorac Surg (2015) 48:684–90. doi: 10.1093/ejcts/ezu504 25567960

[8] Uy KL Darling G Xu W Yi Q-L De Perrot M Pierre AF. Improved Results of Induction Chemoradiation Before Surgical Intervention for Selected Patients With Stage IIIA-N2 Non-Small Cell Lung Cancer. J Thorac Cardiovasc Surg (2007) 134:188–93. doi: 10.1016/j.jtcvs.2007.01.078 17599507

[9] Fukuoka M Wu Y-L Thongprasert S Sunpaweravong P Leong S-S Sriuranpong V. Biomarker Analyses and Final Overall Survival Results From a Phase III, Randomized, Open-Label, First-Line Study of Gefitinib Versus Carboplatin/Paclitaxel in Clinically Selected Patients With Advanced non-Small-Cell Lung Cancer in Asia (IPASS). J Clin Oncol (2011) 29:2866–74. doi: 10.1200/JCO.2010.33.4235 21670455

[10] Wu Y-L Zhou C Hu C-P Feng J Lu S Huang Y. Afatinib Versus Cisplatin Plus Gemcitabine for First-Line Treatment of Asian Patients With Advanced Non-Small-Cell Lung Cancer Harbouring EGFR Mutations (LUX-Lung 6): An Open-Label, Randomised Phase 3 Trial. Lancet Oncol (2014) 15:213–22. doi: 10.1016/S1470-2045(13)70604-1 24439929

[11] Cheng Y He Y Li W Zhang H-l Zhou Q Wang B. Osimertinib Versus Comparator EGFR TKI as First-Line Treatment for EGFR-Mutated Advanced NSCLC: FLAURA China, A Randomized Study. Target Oncol (2021) 16(2):165–76. doi: 10.1007/s11523-021-00794-6 PMC7935816 33544337

[12] Lu S Wang Q Zhang G Dong X Yang CT Song Y. Efficacy of Aumolertinib (HS-10296) in Patients With Advanced EGFR T790M+ NSCLC: Updated Post-National Medical Products Administration Approval Results From the APOLLO Registrational Trial. J Thorac Oncol (2022) 17(3):411–22. doi: 10.1016/j.jtho.2021.10.024 34801749

[13] Romero D. Aumolertinib Is Effective in NSCLC. Nat Rev Clin Oncol (2022) 19(1):6. doi: 10.1038/s41571-021-00586-x 34845385

[14] Lu S Dong X Jian H Chen J Chen G Sun Y. Randomized Phase III Trial of Aumolertinib (HS-10296, Au) Versus Gefitinib (G) as First-Line Treatment of Patients With Locally Advanced or Metastatic Non-Small Cell Lung Cancer (NSCLC) and EGFR Exon 19 Del or L858R Mutations (EGFRm). J Clin Oncol (2021) 39. doi: 10.1200/JCO.2021.39.15_suppl.9013 PMC9509093 35580297

[15] Pujade-Lauraine E Wagner U Aavall-Lundqvist E Gebski V Heywood M Vasey PA. Pegylated Liposomal Doxorubicin and Carboplatin Compared With Paclitaxel and Carboplatin for Patients With Platinum-Sensitive Ovarian Cancer in Late Relapse. J Clin Oncol (2010) 28(20):3323–9. doi: 10.1200/JCO.2009.25.7519 20498395

[16] Zhong WZ Chen KN Chen C Gu CD Wang J Yang XN. Erlotinib Versus Gemcitabine Plus Cisplatin as Neoadjuvant Treatment of Stage IIIA-N2 EGFR-Mutant Non-Small-Cell Lung Cancer (EMERGING-CTONG 1103): A Randomized Phase II Study. J Clin Oncol (2019) 37(25):2235–45. doi: 10.1200/JCO.19.00075 31194613

[17] Zhong W Yang X Yan H Zhang X Su J Chen Z. Phase II Study of Biomarker-Guided Neoadjuvant Treatment Strategy for IIIA-N2 Non-Small Cell Lung Cancer Based on Epidermal Growth Factor Receptor Mutation Status. J Hematol Oncol (2015) 8:54. doi: 10.1186/s13045-015-0151-3 25981169 PMC4455050

[18] Lara-Guerra H Chung CT Schwock J Pintilie M Hwang DM Leighl NB. Histopathological and Immunohistochemical Features Associated With Clinical Response to Neoadjuvant Gefitinib Therapy in Early Stage Non-Small Cell Lung Cancer. Lung Cancer (2012) 76:235–41. doi: 10.1016/j.lungcan.2011.10.020 22112291

[19] Lara-Guerra H Waddell TK Salvarrey MA Joshua AM Chung CT Paul N. Phase II Study of Preoperative Gefitinib in Clinical Stage I Non-Small-Cell Lung Cancer. J Clin Oncol (2009) 27:6229–36. doi: 10.1200/JCO.2009.22.3370 19884551

[20] Hishida T Nagai K Mitsudomi T Yokoi K Kondo H Horinouchi H. Salvage Surgery for Advanced Non-Small Cell Lung Cancer After Response to Gefitinib. J Thorac Cardiovasc Surg (2010) 140:e69-e71. doi: 10.1016/j.jtcvs.2010.06.035 20674944

[21] Schaake EE Kappers I Codrington HE Valdes Olmos RA Teertstra HJ van Pel R. Tumor Response and Toxicity of Neoadjuvant Erlotinib in Patients With Early-Stage non-Small-Cell Lung Cancer. J Clin Oncol (2012) 30:2731–8. doi: 10.1200/JCO.2011.39.4882 22753915

[22] Rice JD Heidel J Trivedi JR van Berkel VH. Optimal Surgical Timing After Neoadjuvant Therapy for Stage IIIa Non-Small Cell Lung Cancer. Ann Thorac Surg (2020) 109(3):842–7. doi: 10.1016/j.athoracsur.2019.09.076 31756320

